# Claudin-19 Mutations and Clinical Phenotype in Spanish Patients with Familial Hypomagnesemia with Hypercalciuria and Nephrocalcinosis

**DOI:** 10.1371/journal.pone.0053151

**Published:** 2013-01-03

**Authors:** Félix Claverie-Martín, Víctor García-Nieto, Cesar Loris, Gema Ariceta, Inmaculada Nadal, Laura Espinosa, Ángeles Fernández-Maseda, Montserrat Antón-Gamero, África Avila, Álvaro Madrid, Hilaria González-Acosta, Elizabeth Córdoba-Lanus, Fernando Santos, Marta Gil-Calvo, Mar Espino, Elena García-Martinez, Ana Sanchez, Rafael Muley, RenalTube Group

**Affiliations:** 1 Unidad de Investigación, Hospital Nuestra Señora de Candelaria, Santa Cruz de Tenerife, Spain; 2 Nefrología Pediátrica, Hospital Nuestra Señora de Candelaria, Santa Cruz de Tenerife, Spain; 3 Nefrología Pediátrica, Hospital Infantil Miguel Servet, Zaragoza, Spain; 4 Nefrología Pediátrica, Hospital de Cruces, Baracaldo, Spain; 5 Nefrología Pediátrica, Hospital Virgen del Camino, Pamplona, Spain; 6 Nefrología Pediátrica, Hospital La Paz, Madrid, Spain; 7 Nefrología Pediátrica, Hospital Virgen de la Salud, Toledo, Spain; 8 Nefrología Pediátrica, Hospital Reina Sofía, Córdoba, Spain; 9 Nefrología Pediátrica, Complejo Hospitalario de Jaén, Jaén, Spain; 10 Nefrología Pediátrica, Hospital Vall d’Hebron, Barcelona, Spain; 11 Nefrología Pediátrica, Hospital Central de Asturias, Oviedo, Spain; 12 Nefrología Pediátrica, Hospital Clínico, Santiago de Compostela, Spain; 13 Pediatría, Hospital Fundación Alcorcón, Madrid, Spain; 14 Nefrología Pediátrica, Hospital Universitario Virgen del Rocío, Sevilla, Spain; 15 Nefrología Pediátrica, Hospital 12 de Octubre, Madrid, Spain; Instituto de Ciencia de Materiales de Madrid - Instituto de Biomedicina de Valencia, Spain

## Abstract

Familial hypomagnesemia with hypercalciuria and nephrocalcinosis is an autosomal recessive tubular disorder characterized by excessive renal magnesium and calcium excretion and chronic kidney failure. This rare disease is caused by mutations in the *CLDN16* and *CLDN19* genes. These genes encode the tight junction proteins claudin-16 and claudin-19, respectively, which regulate the paracellular ion reabsortion in the kidney. Patients with mutations in the *CLDN19* gene also present severe visual impairment. Our goals in this study were to examine the clinical characteristics of a large cohort of Spanish patients with this disorder and to identify the disease causing mutations. We included a total of 31 patients belonging to 27 unrelated families and studied renal and ocular manifestations. We then analyzed by direct DNA sequencing the coding regions of *CLDN16* and *CLDN19* genes in these patients. Bioinformatic tools were used to predict the consequences of mutations. Clinical evaluation showed ocular defects in 87% of patients, including mainly myopia, nystagmus and macular colobomata. Twenty two percent of patients underwent renal transplantation and impaired renal function was observed in another 61% of patients. Results of the genetic analysis revealed *CLDN19* mutations in all patients confirming the clinical diagnosis. The majority of patients exhibited the previously described p.G20D mutation. Haplotype analysis using three microsatellite markers showed a founder effect for this recurrent mutation in our cohort. We also identified four new pathogenic mutations in *CLDN19*, p.G122R, p.I41T, p.G75C and p.G75S. A strategy based on microsequencing was designed to facilitate the genetic diagnosis of this disease. Our data indicate that patients with *CLDN19* mutations have a high risk of progression to chronic renal disease.

## Introduction

Familial hypomagnesemia with hypercalciuria and nephrocalcinosis (FHHNC; MIM #248250) is an autosomal recessive tubular disease characterized by renal magnesium and calcium wasting [1]. FHHNC patients usually present during early childhood or before adolescence with recurrent urinary tract infections, polyuria/polydipsia, nephrolithiasis, and/or failure to thrive [2]–[4]. Complications of hypomagnesemia such as seizures and muscular tetany are less common. Patients develop nephrocalcinosis and chronic kidney failure early in life, and about one third of them develop end-stage renal disease during adolescence. Extrarenal manifestations such as ocular defects have been reported in some cases [1], [4].

In 1999, Simon and colleagues identified mutations in the gene *PCLN1,* subsequently called *CLDN16*, as the underlying cause of FHHNC [5]. Other groups later confirmed this and more *CLDN16* mutations were reported in families with FHHNC [6]–[12]. *CLDN16* encodes claudin-16, a tight junction protein of the claudin multigene family [13]. Claudins are transmembrane proteins with four membrane domains and two extracellular domains that form the paracellular pore and barrier. They play a key role in the regulation and selectivity of paracellular ion reabsortion in the kidney [14]. Konrad and colleagues reported nine families affected with FHHNC that did not have any mutations in *CLDN16* [15]. These families were distinct in that affected individuals showed severe ocular defects including severe myopia, nystagmus, and macular colobomata, features that are not present or appear mildly among patients with *CLDN16* mutations [3], [15]. Another tight junction gene, *CLDN19*, was identified as the cause of the disease in these patients [15]. This type of renal magnesium wasting with hypercalciuria and progressive renal failure has been named FHHNC with severe ocular involvement (MIM #248190). *CLDN19* encodes claudin-19 that is expressed in renal tubules, retina and myelinated peripheral neurons [15]–[17]. In the kidney, claudin-19 colocalizes with claudin-16 at tight junctions of the TAL. Recent work has demonstrated that claudin-16 and claudin-19 require each other for assembly into tight junctions in the TAL [18], [19]. The heteromeric claudin-16 and claudin-19 interaction forms a cation-selective paracellular channel needed for normal levels of magnesium reabsortion. Loss of either claudin-16 or claudin-19 in the mouse kidney abolishes the cation selectivity for the TAL paracellular pathway, leading to excessive renal wasting of magnesium [20].

In the present study, we investigated the clinical characteristics and the genotype in 31 FHHNC Spanish patients. Most patients presented ocular defects and all of them had mutations in *CLDN19*. Haplotype analysis was performed to confirm that p.G20D is a founder mutation. Also, we extended the spectrum of known *CLDN19* mutations by describing four new mutations.

Preliminary reports of this work have been published in abstract form [21], [22].

## Materials and Methods

### Ethics Statement

The Ethics Committee of Hospital Universitario Nuestra Señora de Candelaria approved the present study, and written informed consent for the genetic analysis was obtained from all patients and/or their parents.

### Patient Recruitment and Clinical Evaluation

Thirty-one FHHNC patients from 27 nonconsanguineous families were recruited. All families originated from Spain, except one from Venezuela. The criteria used for the diagnosis of this disease were: hypomagnesemia (except in those cases who had already developed renal failure), hypercalciuria, nephrocalcinosis and absence of metabolic alkalosis [1], [3], [4]. The patients were documented for renal and ocular phenotypic manifestations, and routine parameters were recorded at time of diagnosis. Partial clinical information on patients P203, P206, P207, P211, P216, P316, P317, P333, P343 has been included in a previous report [23]. Serum and urine levels of magnesium and calcium were analyzed using standard laboratory techniques in the central laboratories of the respective hospital. Hypomagnesemia was defined when serum magnesium levels were maintained below 1.6 mg/dl. Urinary calcium levels above 4 mg/kg/day were considered as hypercalciuria. Glomerular filtration rate (GFR) was calculated using the Schwartz formula [24]. GFR levels below 90 ml/min/1,73 m^2^ were considered as chronic kidney disease. Ophthalmologic exploration was performed in the Ophthalmology Services of the respective hospitals. Bilateral nephrocalcinosis was detected by ultrasound or radiography.

### Mutation Screening

Total DNA was extracted from whole blood using the QIAamp DNA Blood Mini Kit (Qiagen, Hilden, Germany). Four primer pairs were designed to PCR amplify the five exons of *CLDN19* (Table S1). The *CLDN16* coding region was amplified by PCR using primers and conditions previously described [5]. PCR products were purified with the QIAquick PCR purification kit (Qiagen) and sequenced with the BigDye Terminator v3.1 Cycle Sequencing Kit (Applied Biosystems, Foster City, CA, USA). Sequence reactions were purified with DyeEx 2.0 Spin columns (Qiagen) before analysis on an ABI PRISM 310 Genetic Analyzer (Applied Biosystems). All PCR products were bidirectionally sequenced. DNA mutations were determined by comparison to reference sequences (GeneBank accession number NG_008993 for *CLDN19*), and confirmed by sequencing the DNA from the respective parents. We analyzed the segregation of mutations with disease by SNaPshot or direct sequencing of DNA from all available family members.

### Bioinformatic Analysis of Mutations

The effect of aminoacid substitutions on protein function was predicted using SIFT, PolyPhen and Align GVGD softwares [25]–[27]. The NCBI reference sequence (NP_683763.2) of the human claudin-19 protein was used as the input for SIFT and PolyPhen, with default query options. A multiple sequence alignment of claudin-19 proteins and other related members of the claudin superfamily in vertebrate species was used as input for Align GVGD. Mutations close to the splice site were evaluated with the NNSPLICE 0.9 version of the *Splice Site Prediction by Neural Network* software [28].

### SNaPshot Extension Reactions

SNaPshot analysis was used to screen the new variants in 102 ethnically matched control subjects (204 chromosomes) and to detect mutations in family members. *CLDN19* exons were amplified by PCR as described above. Unconsumed dNTPs and primers remaining in the PCR product mixture were removed by treatment with ExoSAP-IT (USB, Cleveland, OH, USA). SNaPshot minisequencing was performed with the ABI PRISM®SNaPshot^TM^Multiplex Kit (Applied Biosystems) under the manufacturer’s conditions. Specific primers for each mutation were designed using the SBE primer software v.1.0 (Table S2). After treatment with SAP (USB), the sequencing products were mix with formamide and GeneScan-120 LIZ Size Standard (Applied Biosystems), denatured and resolved using the ABI PRISM 310 Genetic Analyzer (Applied Biosystems). Data were analyzed with the GeneMapper™ 3.7 Software (Applied Biosystems). DNAs from patients with the different mutations were included as positive controls.

### Polymorphic Marker Analysis

To determine whether the p.G20D *ΧΛΔΝ19* mutation derived from a common founder, we performed an haplotype analysis on genomic DNA using microsatellite markers D1S463, D1S193, and D1S447 (GenBank accession numbers Z23403, Z16490.1, and Z23291.1, respectively. NCBI UniSTS database http://www.ncbi.nlm.nih.gov/genome/sts). Polymorphic loci D1S463 and D1S193 are located 0.5 cM and 0.18 cM upstream of *ΧΛΔΝ19*, respectively, and D1S447 is 0.75 cM downstream of *ΧΛΔΝ19*. The microsatellite analysis was carried out as described by Rosa Vargass-Poussou and colleagues [29]. The forward primer for the amplification of each marker was end-labeled with 6-FAM (Fluorescein) (Integrated DNA Technologies, Leuven, Belgium). The PCR products were electrophoresed through a 3500 Series Genetic Analyzer (Applied Biosystems), sized using the GeneScan-500 LIZ Size Standard, and analyzed with the GeneMapper Software version 4.1 (Applied Biosystems).

## Results

### Clinical Phenotypes

The clinical characteristics, biochemical data and genotypes of the 31 patients (19 females and 12 males) included in this study are summarized in Tables 1, 2 and 3. Disease symptoms were detected during the first 1 or 2 years of life in most cases (median 1 year; range 3 months to 20 years). The median age at onset of symptoms was 1 year for male patients and 1.6 years for female patients. First symptoms included urinary tract infections, polyuria/polydipsia, abdominal pain, vomiting and failure to thrive. Bilateral nephrocalcinosis was found in all patients. Recurrent urinary tract infection was a frequent presenting symptom (48% of patients) (Tables 1, 2 and 3). The lowest and highest serum magnesium levels recorded were 0.8 mg/dl and 1.8 mg/dl, respectively. For 15 patients the serum magnesium level was 1.2 mg/dl or lower, and for 14 patients was between 1.2 and 1.6 mg/dl. The median value of the lowest serum magnesium levels was 1.3 mg/dl. Two patients showed normal serum magnesium levels, due to the fact that at diagnosis they already had chronic renal disease. Renal loss of calcium and magnesium was also found in all patients except in those cases where renal function was deteriorated (Tables 1, 2 and 3). In several cases, the levels of urinary calcium excretion and urinary magnesium excretion were not determined as they could be affected by chronic renal disease. The median maximal urinary calcium excretion value was 10.6 mg/kg per day, with a range of 3.1 to 16.6 mg/kg per day. The actual GFR filtration rate was >90 ml/min per 1.73 m^2^ for 5 affected children (age range of 2.8 to 11 years), 60 to 89 ml/min per 1.73 m^2^ for 8 patients (age range of 6.6 to 19.6 years), 30 to 59 ml/min per 1.73 m^2^ for 6 patients (age range of 5.9 to 27.8 years), and <30 ml/min per 1.73 m^2^ for 5 patients (age range of 3.5 to 34 years, two of them on chronic peritoneal dialysis). Seven patients (age range of 7.8 to 48 years), all females, underwent kidney transplantation. Different ocular defects were observed in 27 patients (16 females and 11 males) (87%), including mainly myopia, nystagmus, and macular colobomata, and also chorioretinitis, macular scar, hypermetropia, macular degeneration, hypopigmented retina and astigmatism (Tables 1, 2 and 3). Patient P393 showed tremors upon waking and also difficulty for fine psycomotricity and, in occasions, gait instability. However, these neurological symptoms have improved over time.

**Table 1:** Clinical characteristics and *CLDN19* mutations of patients with FHHNC with severe ocular defects.

*Family*	*F1* *	*F2* *	F3*	F4*	F4*	F5*	F6*	F6*	F7*	F8*
***Patient***	***P396***	***P393***	**P203**	**P206**	**P207**	**P211**	**P316**	**P317**	**P333**	**P216**
**Gender**	F	M	M	F	M	M	F	F	F	F
**Age at onset** a	2	0.4	0.5	3	1	0.8	0.3	2.5	1	0.5
**Present age** b	21.2	6.8	11	19.6	12.1	13.5	40	32	48	44
**Plasma Mg^2+^** c	0.8	1.2	1.2	1.4	1.5	1.5	1.1	1.2	0.8	1.1
**Urinary Ca^2+^** d	7	13.2	12	8.8	7	8.5	14	16	9	12
**Urinary Mg^2+^** d	ND	ND	6.2	5.2	3.4	4.2	6.1	4.5	5	2.7
**Nephrocalcinosis**	+	+	+	+	+	+	+	+	+	+
**UTI** e	+	–	+	–	–	–	+	–	–	+
**Actual renal function** f	44.6	86.6	93	85	83	82	RT	RT	RT	RT
**Ocular phenotype** g	Ma	Normal	N	Ma	N Ma	MC N Ma	MC N Ma	Ma	MC N Ma	MC N Ma
***CLDN19*** ** mutation**	p.G20D	p.G20D	p.G20D	p.G20D	p.G20D	p.G20D	p.G20D	p.G20D	p.G20D	p.G20D
	p.G20D	p.G20D	p.G75C	p.G75S	p.G75S	p.G20D	p.G20D	p.G20D	p.G20D	p.G20D

Listed are the means of all available results for each patient. F, female; M, male; ND, not determined; RT, renal transplant; +, present; −, absent.

a Age at onset of symptoms in years;

b In years;

c In mg/dl;

d In mg/kg per day;

e Urinary tract infections;

f GFR, ml/min per 1.73 m^2^;

g N, Nystagmus; Ma, Myopia; MC, Macular colobomata.

* Families in which segregation of mutations was demonstrated. Both parents were shown to be heterozygous for the respective mutations except in families F7 and F8 in which only the mother’s sample was available.

**Table 2:** Clinical characteristics and *CLDN19* mutations of patients with FHHNC with severe ocular defects.

*Family*	*F9* *	*F10*	*F11*	F12*	F12*	F13	F14	F15	F16	F17
***Patient***	***P343***	***P243***	***P298***	**P291**	**P439**	**P297**	**P389**	**P388**	**P377**	**P410**
**Gender**	F	M	M	F	F	F	F	F	F	F
**Age at onset** a	1	8	2	0.9	0.7	1	0.3	10	0.7	1.9
**Present age** b	35.3	27.8	14.7	7.8	5.7	20.1	6	19	7	7.9
**Plasma Mg^2+^** c	1.4	0.8	1.4	1.5	1.8	**1.1**	1.2	1.1	1.3	1.2
**Urinary Ca^2+^** d	9	3.15	10.2	ND	ND	15	14.8	11	15.5	4.2
**Urinary Mg^2+^** d	6.3	3.1	4.5	ND	*ND*	8	6	3.2	6.7	4.9
**Nephrocalcinosis**	+	+	+	+	+	+	+	+	+	+
**UTI** e	+	–	–	+	+	+	+	+	–	+
**Actual renal function** f	RT	58.6	34.5	RT	CPD	RT	97	88	66	85
**Ocular phenotype** g	Ma	MC Ma	CR	Normal	Normal	Ma	MC	Ma N MS	N	Normal
***CLDN19*** ** mutation**	p.G20D	p.G122R	p.G20D	p.G20D	p.G20D	p.G20D	p.G20D	p.G20D	p.G20D	p.G20D
	p.G20D	p.G122R	p.G20D	–	–	p.G20D	p.G20D	p.G20D	p.G20D	p.G20D

Listed are the means of all available results for each patient. F, female; M, male; ND, not determined; RT, renal transplant; CPD, chronic peritoneal dialysis; +, present; −, absent.

a Age at onset of symptoms in years;

b In years;

c In mg/dl;

d In mg/kg per day;

e Urinary tract infections;

f GFR, ml/min per 1.73 m^2^;

g N, Nystagmus; Ma, Myopia; MC, Macular colobomata; CR, Chorioretinitis; MS, Macular scar.

* Families in which segregation of mutations was demonstrated. Both parents were shown to be heterozygous for the respective mutations.

**Table 3:** Clinical characteristics and *CLDN19* mutations of patients with FHHNC with severe ocular defects.

*Family*	F18*	F19*	F20*	F21*	F22*	F23*	F24*	F25	F26*	F27*	F27*
***Patient***	**P406**	**P415**	**P416**	**P429**	**P494**	**P451**	**P462**	**P444**	**P455**	**P463**	**P464**
**Gender**	F	F	M	F	M	M	F	F	M	M	M
**Age at onset^a^**	3.4	0.3	14	3	1.2	10.8	8.4	1	20	0.7	2.9
**Present age** b	8.5	6.6	18.7	8	2.8	14	11	5.5	34	3.5	5.9
**Plasma Mg^2+^** c	1.4	1.5	0.9	1.2	1.6	1.5	1.6	1.8	1.4	1.4	1.2
**Urinary Ca^2+^** d	16.6	16.1	8.8	8.2	14.8	5	8.6	7.6	ND	7	11.2
**Urinary Mg^2+^** d	3.6	2.6	2.2	7.5	6.2	ND	3.4	5.6	ND	6	5.4
**Nephrocalcinosis**	+	+	+	+	+	+	+	+	+	+	+
**UTI** e	–	–	–	–	–	+	+	–	–	+	+
**Actual renal function** f	138.2	60	25	96.7	92.1	30	39	CPD	16	18	50
**Ocular phenotype** g	Ma A	Ma	Ma CR	Ma	Ma, HR	Ma	Ma	MC H	Ma MD	Ma	MD
***CLDN19*** ** mutation**	p.G20D	p.G20D	p.G20D	p.Q57E	p.G20D	p.G20D	p.G20D	p.G20D	p.G20D	p.G20D	p.G20D
	p.I41T	p.G20D	p.G20D	p.Q57E	p.G20D	p.G20D	p.G20D	p.G20D	p.G20D	p.G20D	p.G20D

Listed are the means of all available results for each patient. F, female; M, male; ND, not determined; CPD, chronic peritoneal dialysis; +, present; −, absent. ^a^Age at onset of symptoms in years;

b In years;

c In mg/dl;

d In mg/kg per day;

e Urinary tract infections;

f GFR, ml/min per 1.73 m^2^;

g Ma, Myopia; MC, Macular colobomata; CR, Chorioretinitis; H, hypermetropia; MD, macular degeneration; HR, hypopigmented retina; A, astigmatism.

* Families in which segregation of mutations was demonstrated. Both parents were shown to be heterozygous for the respective mutations.

Patients were treated with thiazide diuretics and magnesium salts until they had severe chronic kidney disease. Nevertheless, these treatments did not have a considerable effect on the levels of urinary calcium or serum magnesium (results not shown) in agreement with previous reports [3], [12], [29], [30]. In the kidney transplant patients, calcium and magnesium excretion was normalized.

### Mutations in the Claudin-19 Gene

**Figure 1:**
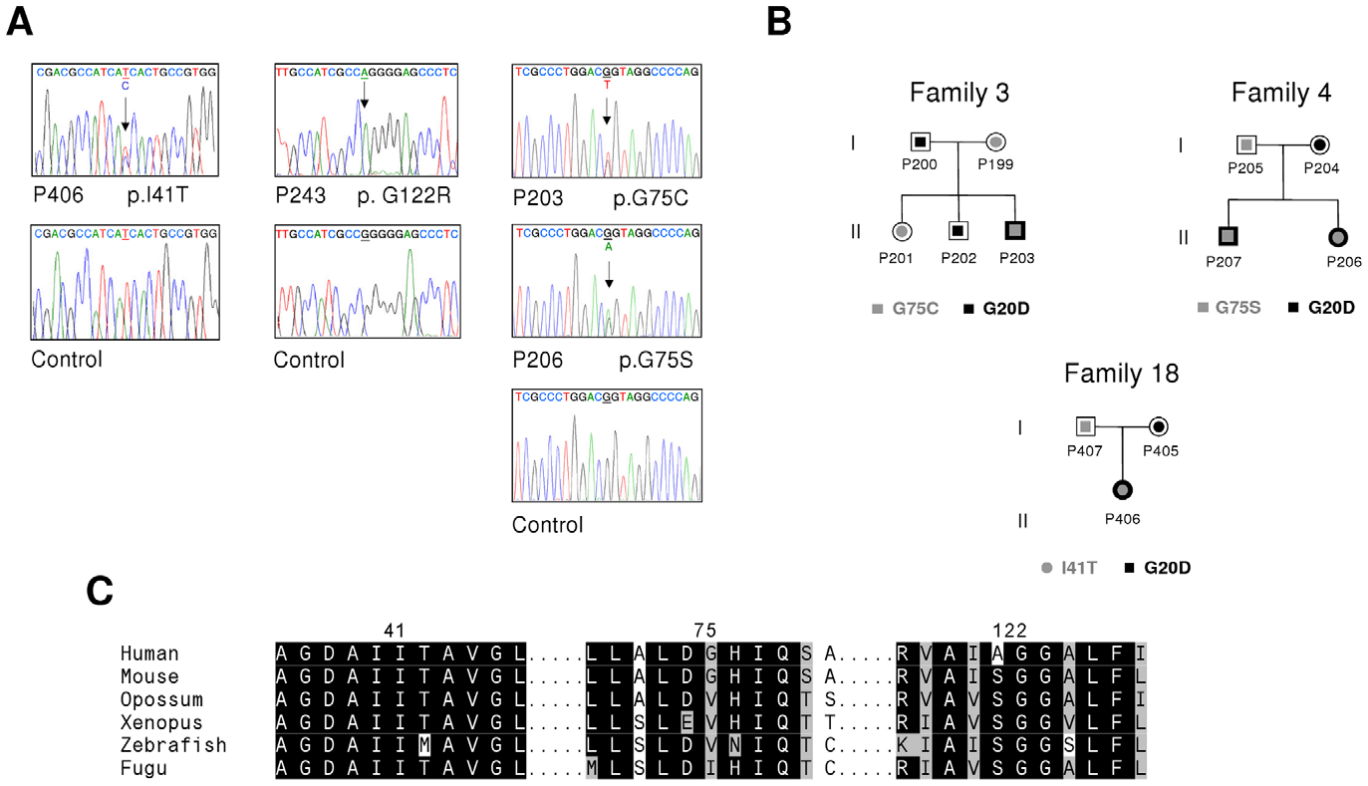
*CLDN19* sequence analysis, pedigrees of three families and multiple sequence alignments. (A) Electropherograms showing homozygous p.G122R mutation in patient P243, mutations p.I41T, p.G75C, p.G75S in heterozygous state in patients P406, P203 and P206, respectively, and controls (bottom panels). The arrows indicate the nucleotide positions with the changes. (B) Pedigrees of families 3, 4 and 18 with genotypes showing 4 compound heterozygous patients, heterozygous parents and two heterozygous siblings. (C) The amino acid sequence alignment of claudin-19 in six species (*Homo sapiens, Mus musculus, Monodelphis domestica, Xenopus laevis, Danio rerio and Takifugu rubripes*) for the new variants identified. p.I41T and p.G122R are in completely conserve positions in claudin-19 orthologs to Fugu, while p.G75C and p.G75S are in a moderately conserved position.

**Figure 2:**
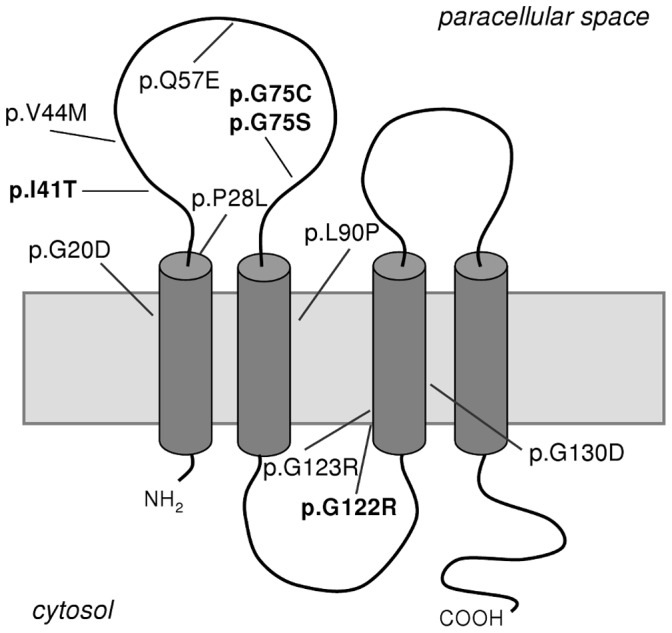
Schematic representation of the claudin-19 protein and localization of missense *CLDN19* mutations. Novel mutations identified in this study are in boldface.

Mutational analysis by direct sequencing of *CLDN19* exons revealed mutations in all patients. *CLDN16* mutations were not detected. Six different *CLDN19* point mutations were identified, two of which, p.G20D and p.Q57E, had been previously described [15], and four were novel, p.I41T (c.122T>C), p.G75C (c.223G>T), p.G75S (c.223G>A) and p.G122R (c.364G>A) (Tables 1, 2 and 3, Figure 1A). These mutations were located in exon 1, except p.G122R, which was located in exon 2. The location of these mutations on the schematic model of claudin-19 is indicated in Figure 2. Both mutant *CLDN19* alleles were detected in all but two affected patients. Twenty-three affected patients were homozygous for p.G20D, and two affected sisters, P291 and P439, were heterozygous for this mutation (Tables 1, 2 and 3). Two affected patients, P243 and P429 were homozygous for mutations p.G122R and p.Q57E, respectively. Parental consanguinity was not reported in these two families but we cannot rule out this possibility. Another four patients, P206, P207, P203 and P406 were compound heterozygous p.G20D/p.G75S, p.G20D/p.G75S, p.G20D/p.G75C and p.G20D/p.I41T, respectively. Mutation p.G20D was found in a total of 29 patients out of 31 (Tables 1, 2 and 3). Using the SNaPshot extension procedure or DNA sequencing, as described in Materials and Methods, we also analyzed 61 nonaffected family members. We confirmed that the mutations were inherited from the patients’ parents in a total of 19 families (Figure 1B, Figure S1 and results not shown). In 10 families we identified another 19 mutation carriers. We then used a multiplex SNaPshot reaction to analyze the altered positions p.I41T, p.G75C, p.G75S and p.G122R, and showed that none of these new variants are found in the 204 ethnically matched control chromosomes (Figure S1 and results not shown). Furthermore, the new alterations were not found in the 1000 Genomes Project (TGP) data or in the NCBI SNP database (dbSNP). Another SNaPshot reaction was designed to detect the p.G20D mutation in different family members and the results were then confirmed by DNA sequencing (Figure S1 and results not shown).

### Potential Deleterious Effects of Mutations

The novel *CLDN19* substitution c.364G>A, found homozygous in patient P243 (Figure 1A), was predicted to change glycine to arginine at residue 122 (p.G122R), a nonconservative amino acid substitution. This particular glycine residue in the third transmembrane domain of claudin-19 is completely conserved in claudin-19 orthologs ranging from humans through mammals and amphibian (Figures 1C and 2). To more rigorously test the significance of this substitution we used three computer tools (SIFT, PolyPhen, and AlignGVGD). Evaluation of p.G122R showed that the mutation was predicted to be damaging (Table 4). The novel substitution, c.122T>C; I41T, found heterozygous in patient P406 (Figure 1A and 1B) compound with G20D, affects the first extracellular loop of claudin-19 (Figure 2). It is also a nonconsevative change of isoleucine to threonine in residue I41 which is highly conserved throughout evolution (Figure 1C). Analysis with the prediction tools indicated that it is most likely a pathogenic change (Table 4). The other two new mutations, c.223G>T (p.G75C) and c.223G>A (p.G75S), predict the change of glycine 75, located in the first extracellular domain of claudin-19, for cysteine and serine, respectively (Figure 2). Both aminoacid substitutions are nonconservative, but aminoacid residue 75 is not so well conserved in evolution (Figure 1C). Evaluation of both p.G75C and p.G75S with the computer tools SIFT, PolyPhen, and AlignGVGD showed that they were predicted to be benign aminoacid substitution that do not alter protein function (Table 4). However, when studied at the mRNA level, mutations c.223G>T and c.223G>A substitute the last nucleotide, G, of exon 1 for a T and an A, respectively, and therefore, altering the intron 1 donor splice site (Figure S2). We therefore analyzed these two mutant sequences using the splice site prediction program NNSPLICE version 0.9 [28] which showed that the score of the donor splice site was strongly diminished from 0.74 (normal site) to 0.01 (mutants). This predicted the activation of a cryptic donor splice site (score 0.95) in intron 1 downstream from the authentic donor site. The resulting mRNA would contain the first 495 nucleotides of intron 1 that introduce a premature stop codon (Figure S2).

**Table 4:** Scoring of new *CLDN19* mutations for likely pathogenicity.

	SIFT^1^		PolyPhen^2^		Align GVGD^3^		
Mutation	Prediction	Score	Prediction	Score	Prediction	Score	Consenseus
p.I41T	Damaging	0.01	Benign	0.98	Deleterious	C65	Pathogenic
p.G75C	Tolerated	0.06	Possibly damaging	1.61	Unclassified	C15	Neutral
p.G75S	Tolerated	0.91	Benign	0.06	Neutral	C0	Neutral
p.G122R	Damaging	0.01	Possibly damaging	2.11	Deleterious	C65	Pathogenic

1 http://sift.bii.a-star.edu.sg.

2 http://genetics.bwh.harvard.edu/pph.

3 http://agvgd.iarc.fr/agvgd_input.

### The Common Mutation p.G20D in *ΧΛΔΝ19* is a Founder Allele

**Figure 3:**
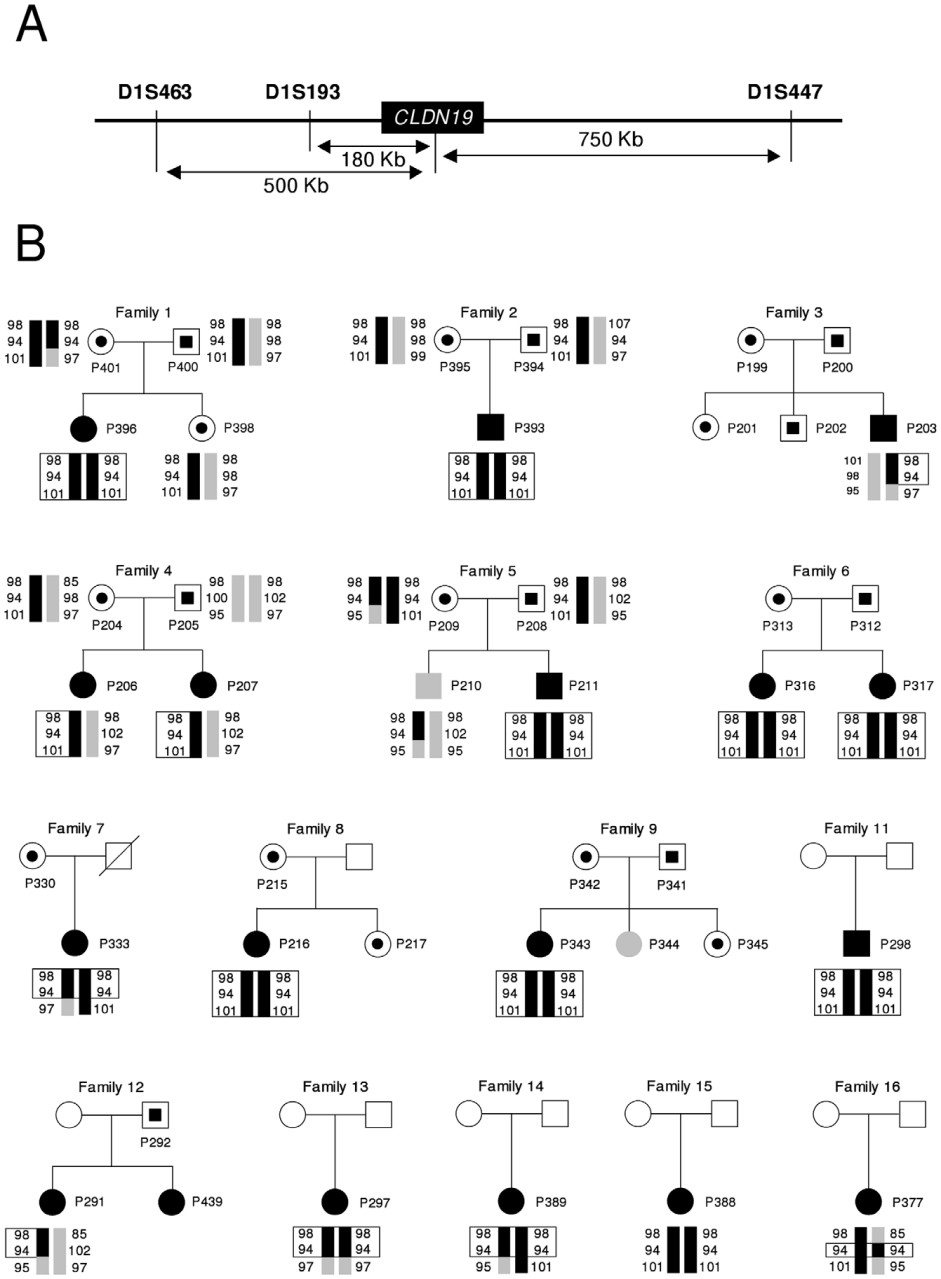
Analysis of three polymorphic microsatellite markers flanking the *CLDN19* locus reveal a founder effect for the common p.G20D mutation. (A) The chromosomal region containing the *CLDN19* locus is shown. Arrows indicate the distances of microsatellite markers from the location of the *CLDN19* mutation. (B) Pedigrees and haplotypes of FHHNC Spanish families 1 to 9 and 11 to 16. Haplotypes for markers D1S463, D1S193 and D1S447 (top to bottom) are represented by vertical bars. The disease-associated alleles are shown in black and the others in gray. Numbers indicate the allele size in nucleotides. Black circles and squares represent affected subjects; black circles and squares within open frames represent heterozygous individuals; gray symbols represent individuals without mutation; and unfilled symbols represent family members who were not analyzed. Squares represent males and circles females. The p.G20D mutation was heterozygous in patients of families 3, 4 and 12, and homozygous in the rest of the patients.

**Figure 4:**
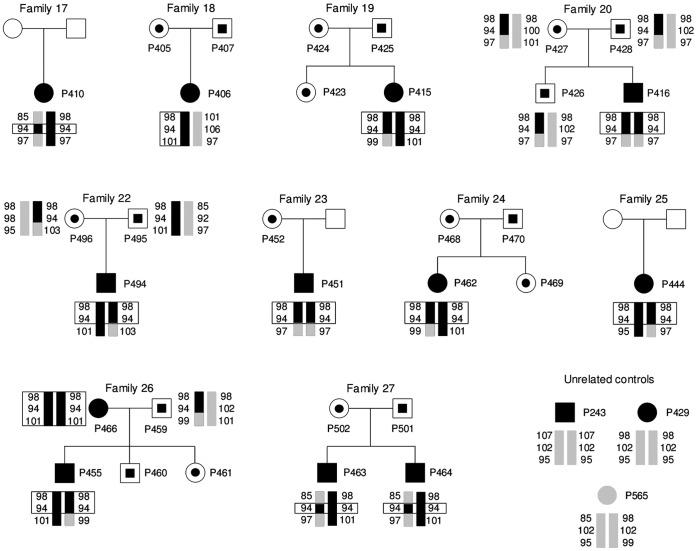
Analysis of three polymorphic microsatellite markers flanking the *CLDN19* locus reveal a founder effect for the common p.G20D mutation. Pedigrees and haplotypes of FHHNC Spanish families 17 to 20 and 22 to 27. Haplotypes for markers D1S463, D1S193 and D1S447 (top to bottom) are represented by vertical bars. The disease-associated alleles are shown in black and the others in gray. Numbers indicate the allele size in nucleotides. Black circles and squares represent affected subjects; black circles and squares within open frames represent heterozygous individuals; gray symbols represent individuals without mutation; and unfilled symbols represent family members who were not analyzed. Squares represent males and circles females. The p.G20D mutation was heterozygous in the patient of family 18, and homozygous in the rest of the patients. Three unrelated individuals, P243, P429 and P565, were included as controls; P243 and P429 are homozygous for mutations G122R and Q57E, respectively, and P565 is a healthy control. The p.G20D-associated haplotype was absent in these controls.

In our study, p.G20D was a recurrent *ΧΛΔΝ19* mutation in the Spanish cohort (Tables 1, 2 and 3). To determine whether this recurrent mutation has arisen from a common ancestor, haplotype analysis was performed in all the patients. The genotypes of patients, some of their relatives and unrelated controls were examined for the three polymorphic microsatellite markers flanking the *ΧΛΔΝ19* locus. This allowed us to determine the disease haplotype that cosegregated with FHHNC in Spanish families. Our results revealed the presence of a mutation-associated haplotype in the patients with the p.G20D mutation, especially at the D1S193 marker (Figures 3 and 4), suggesting remote consanguinity and a founder effect. This haplotype was not observed in the unrelated control and was absent in the patients with the p.G122R or the p.Q57E mutation (Figure 4).

## Discussion

We report clinical and molecular findings about a cohort of 31 patients affected with FHHNC. Genetic analysis of the patients’ DNA revealed 6 different *CLDN19* mutations, four of which are novel. Interestingly, none of the patients showed mutation in the *CLDN16* gene. These results agree with those of Vargas-Poussou and colleagues, who reported *CLDN19* mutations in the 2 Spanish FHHNC patients and the 17 patients from southwest France included in the study [29]. On the contrary, most of their patients from North Africa had mutations in the *CLDN16* gene but not in *CLDN19*. Another 7 Spanish/Hispanic FHHNC patients previously characterized presented only *CLDN19* mutations [15]. As far as we know, only one Spanish patient presented a mutation in the *CLDN16* gene [9]. From these results we can conclude that FHHNC in Spain and southwest France is mainly caused by *CLDN19* mutations. In our study, mutation p.G20D was detected in 93.5% of patients, mostly in homozygous state. This mutation has been previously identified by Konrad and colleagues in 7 Spanish/Hispanic families and referred to as the Spanish/Hispanic mutation [15]. These authors concluded that a founder effect was responsible for the consistency of this mutation in these Spanish/Hispanic families. Recently, Vargas-Poussou and colleagues also detected a founder effect for the *CLDN19* p.G20D mutation in Spanish and French families [29]. In the present study, we demonstrated that the clustering of p.G20D in the Spanish FHHNC cohort is due to a common founder ancestor. Most likely, p.G20D originated from a single mutation event that occurred many generations ago and all these families are distantly related and inherited the mutation from the same individual. Expression analysis have shown that p.G20D causes retention of the mutant protein inside the cell instead of its normal targeting to the cell membrane [15], [18]. Regarding the two patients with the p.G20D mutation in only one allele, the other mutation could be located in a promoter or regulatory region or deep within an intron, or alternatively could be a large deletion and thus may have escaped detection by our approach. One of our patients presented mutation p.Q57E, which has also been previously identified and referred to as the Swiss mutation [15]. This missense mutation results in a complete loss of claudin-19 function [18].

Although no functional studies were performed, the amino acid changes p.I41T and p.G122R, identified in two of our patients, are very likely to be pathogenic mutations based on evolutionary conservations, absence in healthy controls, as well as prediction software (Figure 1C, Table 4). We suggest that p.G122R has a similar effect to that of p.G123R, which has been previously identified and characterized [18]. Mutation p.G123R localizes properly in the tight junction but causes partial claudin-19 loss of function. Protein interaction assays showed that mutation p.G123R severely affects interactions of claudin-19 with claudin-16 and abolishes their synergistic effect in cation selectivity [18]. The other two sequence variations identified in the present study, p.G75C and p.G75S, predict amino acid changes in positions that are less conserved in evolution and that are tolerated or neutral. These two new variations are also absent in healthy controls. The location of these two mutations in the last nucleotide (G) of *CLDN19* exon 1 suggested an effect on pre-mRNA splicing. This nucleotide is actually part of the classical consensus donor splice site and mutations within this motif result in defective mRNAs [31], [32]. Unfortunately no RNA samples were available during the course of this study to analyze the cDNA product. Results obtained with the NNSPLICE software showed that mutations p.G75C and p.G75S lead to a 74-fold decrease in the donor splice site efficiency and to the synthesis of an aberrant mRNA containing a premature stop codon. Transcripts containing premature termination codons are degraded rapidly by the nonsense-mediated mRNA decay pathway [33]. Therefore, these two mutations were predicted to result loss of function, although none of these predictions have been confirmed.

Our clinical data indicate that FHHNC patients with *CLDN19* mutations have a high risk of progression to chronic renal disease. The recent study by Vargas-Possou and colleagues concluded that patients with *CLDN19* mutations may present more severe renal impairment than patients with *CLDN16* mutations [29]. Additionally, Konrad and colleagues have established a genotype-phenotype correlation with regard to the progression of renal failure in FHHNC patients with *CLDN16* mutations [34]. In our study, 61% of the patients were female which is similar to the 58% found by Vargas-Poussou and colleagues in their patients with *CLDN19* mutations. Remarkably, all the transplanted patients in our study were female. Although the disease symptoms were detected during the first 1 or two years of life in most of our patients, four of them remained asymptomatic until 8 to 10 years of age of old, one patient until 14 years of age and one until 20 years of age. Faguer and colleagues [35] have also reported a patient with the homozygous p.G20D *CLDN19* mutation who was asymptomatic until 16 years of age. Therefore, as they concluded, the disease symptoms may go undetected until the second decade of life. Alternatively, this difference could be due to possible genetic modifiers, although we do not have any indication for this. In the present study, we could not establish genotype-phenotype correlations since p.G20D was the only mutation found in high frequency.

Approximately 87% of the patients studied herein had severe ocular defects including myopia, nystagmus and macular colobomata (Tables 1, 2 and 3). This finding is similar to those reported for other series [15], [29]. Therefore, we can conclude that these ocular abnormalities are characteristics of FHHNC patients with *CLDN19* mutations. Four of our patients (ages between 5.7 and 7.9) did not present ocular abnormalities. Two of these patients (6.8 and 7.9 years old) were homozygous for p.G20D and two sisters (5.7 and 7.8 years old) were heterozygous for this mutation. One of the Spanish patients with mutation p.G20D, F51-II-1, studied by Konrad and his associates [15], also persisted at 16 years of age without ocular lesions. We suggest that there must be other unknown factors associated with the ocular phenotype. Claudin-19 is expressed at high levels in the retinal pigment epithelium linking dysfunction of claudin-19 to the phenotype of visual impairment [15]. Claudin-19 is also expressed in the tight junction of Schwann cells where it could participate in electrical insulation of axons [16]. Claudin-19-deficient mice exhibited behavioral abnormalities that could be due to peripheral nervous system deficit [16]. In a recent study, Faguer and colleagues [35] reported muscular-exercise intolerance with cramps, weakness and electromyographical alterations in two FHHNC patients with severe ocular defects. We observed no neuromuscular symptoms in our patients with the exception of patient P393 who showed tremors upon waking, difficulty for fine psycomotricity and, sporadically, gait instability.

Our patients were treated with thiazides and magnesium salts until they had severe deterioration of renal function. However, these treatments did not have a considerable effect on the levels of urinary calcium or serum magnesium. A recent study treated FHHNC patients with magnesium supplements or thiazide diuretics and found that neither treatment seem to have effect on hypercalciuria or chronic kidney disease progression [29]. Kidney transplantation has been the only effective approach reducing the extensive magnesium and calcium wasting in FHHNC patients since the molecular defect is located in the kidney [1]. As expected, in our kidney transplant patients, calcium and magnesium excretion was normalized.

In conclusion, we examined a large cohort of Spanish patients with FHHNC and described their clinical characteristics and genotype. All patients had mutations in the *CLDN19* gene and the majority presented severe ocular anomalies. These patients have a high risk of progression to chronic renal disease. The recurrent p.G20D mutation found in our cohort is probably due to a founder effect. Also, we identified four novel *CLDN19* mutations that are rare variants with all the characteristics of pathogenic changes. A simple and specific method was developed to facilitate the molecular diagnosis of the disease in the Spanish population.

## Supporting Information

Figure S1 **Detection of** ***CLDN19*** **mutations using microsequencing.** Panels A and B show the results of the GenScan analysis with the multiplex SNaPshot reactions for the simultaneous detection of mutations p.I41T, p.G75S, p.G75C and p.G122, and with the reactions for the detection of mutation p.G20D, respectively. (A) Analysis of samples from patients P203, P206, P243 and P406; red peak p.G75C, green peaks p.G75S, red peak p.G122R and black peak p.I47T (all in bold face) correspond to mutant alleles (T, A, T and C) from patients P203, P206, P243 and P406, respectively. Blue peaks p.G75, red peaks p.I41 and black peaks p.G122 correspond to the incorporation of the normal nucleotide (G, T or C) to the reaction product, respectively (see control). (B) Analysis of patients P206 and P211; black (G20) and red (G20D, bold face) peaks correspond to normal (C) and mutant alleles (T), respectively.
(PDF)

Figure S2 **Predicted effect of** ***CLDN19*** **mutations at mRNA level.** Diagram of the effect of mutations p.G75C and p.G75S on *CLDN19* pre-mRNA splicing, as determined with the NNSPLICE software (http://www.fruitfly.org/seq_tools/splice.html). The region spanning from exon 1 to exon 2 is shown. The arrowhead indicates the location of the activated cryptic donor splice site in intron 1 (thick line). A segment of this intron is included in the altered mature mRNA (top). The correct splice transcript is shown at the bottom. The red arrow indicates the location of the premature stop codon (TGA) in intron 1.
(PDF)

Table S1 **Primers used for PCR amplification and sequencing of** ***CLDN19*** **exons.**
(DOC)

Table S2 **Primers used for minisequencing reactions.**
(DOC)

Appendix S1 **Members of the RenalTube Group**
(DOC)
