# Correction: Claudin-19 Mutations and Clinical Phenotype in Spanish Patients with Familial Hypomagnesemia with Hypercalciuria and Nephrocalcinosis

**DOI:** 10.1371/annotation/25732fb0-ae38-40f6-b8c6-eb4ba94ac996

**Published:** 2013-10-04

**Authors:** Félix Claverie-Martín, Víctor García-Nieto, Cesar Loris, Gema Ariceta, Inmaculada Nadal, Laura Espinosa, Ángeles Fernández-Maseda, Montserrat Antón-Gamero, África Avila, Álvaro Madrid, Hilaria González-Acosta, Elizabeth Córdoba-Lanus, Fernando Santos, Marta Gil-Calvo, Mar Espino, Elena García-Martinez, Ana Sanchez, Rafael Muley, RenalTube Group

All instances of ΧΛΔΝ19 should be read as CLDN19 throughout the "Materials and Methods" and "Results" sections. 

