# Supplementary figures and images for: Claudin-19 Mutations and Clinical Phenotype in Spanish Patients with Familial Hypomagnesemia with Hypercalciuria and Nephrocalcinosis
Source: PLoS One. 2013 Jan 3;8(1):e53151. doi: 10.1371/journal.pone.0053151 (PMC3536807; doi:10.1371/journal.pone.0053151)

Figure S1

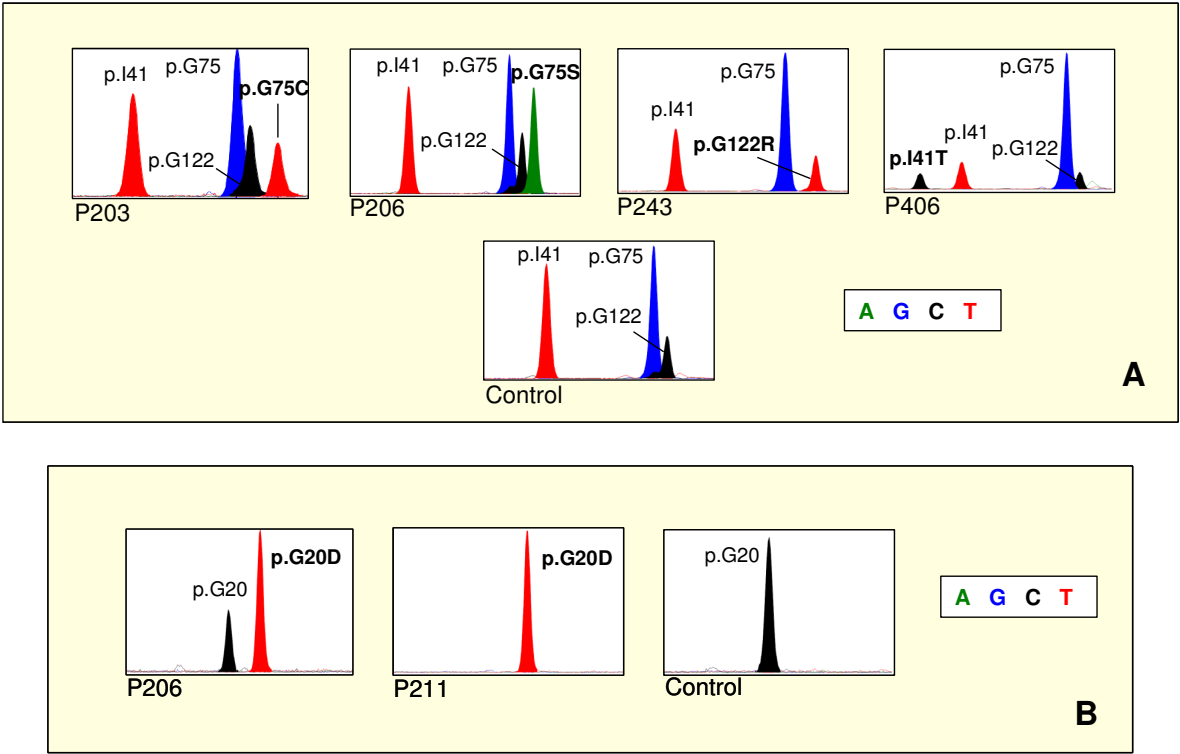

Supplement: Figure S1 — Detection of CLDN19 mutations using microsequencing. Panels A and B show the results of the GenScan analysis with the multiplex SNaPshot reactions for the simultaneous detection of mutations p.I41T, p.G75S, p.G75C and p.G122, and with the reactions for the detection of mutation p.G20D, respectively. (A) Analysis of samples from patients P203, P206, P243 and P406; red peak p.G75C, green peaks p.G75S, red peak p.G122R and black peak p.I47T (all in bold face) correspond to mutant alleles (T, A, T and C) from patients P203, P206, P243 and P406, respectively. Blue peaks p.G75, red peaks p.I41 and black peaks p.G122 correspond to the incorporation of the normal nucleotide (G, T or C) to the reaction product, respectively (see control). (B) Analysis of patients P206 and P211; black (G20) and red (G20D, bold face) peaks correspond to normal (C) and mutant alleles (T), respectively. (PDF) [file pone.0053151.s001.pdf]

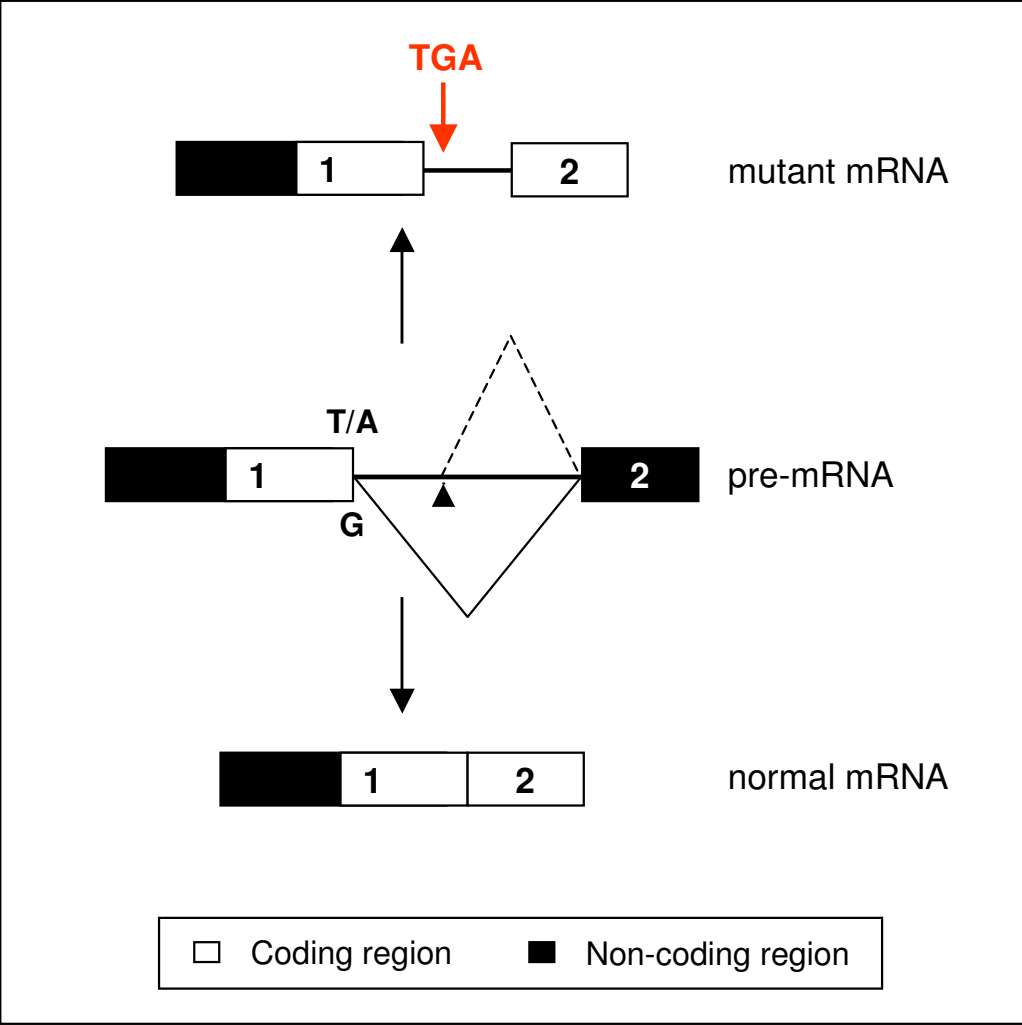

Supplement: Figure S2 — Predicted effect of CLDN19 mutations at mRNA level. Diagram of the effect of mutations p.G75C and p.G75S on CLDN19 pre-mRNA splicing, as determined with the NNSPLICE software (http://www.fruitfly.org/seq_tools/splice.html). The region spanning from exon 1 to exon 2 is shown. The arrowhead indicates the location of the activated cryptic donor splice site in intron 1 (thick line). A segment of this intron is included in the altered mature mRNA (top). The correct splice transcript is shown at the bottom. The red arrow indicates the location of the premature stop codon (TGA) in intron 1. (PDF) [file pone.0053151.s002.pdf]
