# Supplementary material for: Claudin-19 Mutations and Clinical Phenotype in Spanish Patients with Familial Hypomagnesemia with Hypercalciuria and Nephrocalcinosis
Source: PLoS One. 2013 Jan 3;8(1):e53151. doi: 10.1371/journal.pone.0053151 (PMC3536807; doi:10.1371/journal.pone.0053151)
Supplement: Table S1 — Primers used for PCR amplification and sequencing of CLDN19 exons. (DOC) [file pone.0053151.s003.doc]

**Table S1**. **Primers used for PCR amplification and sequencing of *CLDN19* exons**.

| Exon | **Primer sequence (5’-3’)** | | **Annealing**  **temperature (°C)** | **Product size (bases)** |
| --- | --- | --- | --- | --- |
|  | Forward | Reverse |  |  |
| 1 | CACCACCTCTCCTTCTCTGC | CTGTTCCCACCTCCCATCT | 63 | 412 |
| 2 - 3 | GCTGTCACTCCTCACTATCC | GGTAAAGCAAAAGACCCAAG | 55 | 552 |
| 4 | CCACACCTGATGCCACTCT | CCCATCCTATGCCCCAGT | 60 | 298 |
| 5 | CCACAGCTCACTCCACTAACC | ACCCTGGACCTCTGTCTCCT | 63 | 301 |

Internal primer CLD19E1bF (5’-TGCCACAGTGGAAGCAGTCTT-3’) was used in some cases to sequence the 3’

end of exon 1.
