# Supplementary material for: Claudin-19 Mutations and Clinical Phenotype in Spanish Patients with Familial Hypomagnesemia with Hypercalciuria and Nephrocalcinosis
Source: PLoS One. 2013 Jan 3;8(1):e53151. doi: 10.1371/journal.pone.0053151 (PMC3536807; doi:10.1371/journal.pone.0053151)
Supplement: Table S2 — Primers used for minisequencing reactions. (DOC) [file pone.0053151.s004.doc]

**Table S2**. **Primers used for minisequencing reactions.**

| Primer sequence (5’-3’)* | Position | Incorporated nucleotide |
| --- | --- | --- |
| GCTGTGCTAGCAATGATG | G20 | C (normal) or T (mutant) |
| CAGGCGACGCCATCA | I41 | T(normal) or C (mutant) |
| **CCCCCC**TGCTCGCCCTGGAC | G75 | G (normal) or A/T (mutants) |
| **CCCCCCCC**GATGAAGAGGGCTCCCC | G122 | C (normal) or T (mutant) |

*The first and last primers correspond to the noncoding DNA strand, and the other two correspond to the

coding strand. C tails (in bold) were added to two of the primers
