# Supplementary material for: Claudin-19 Mutations and Clinical Phenotype in Spanish Patients with Familial Hypomagnesemia with Hypercalciuria and Nephrocalcinosis
Source: PLoS One. 2013 Jan 3;8(1):e53151. doi: 10.1371/journal.pone.0053151 (PMC3536807; doi:10.1371/journal.pone.0053151)
Supplement: Appendix S1 — Members of the RenalTube Group (DOC) [file pone.0053151.s005.doc]

**Appendix S1 RenalTube Group Investigators**

1. Unidad de Investigación y Unidad de Nefrología Pediátrica, Hospital Universitario Nuestra Señora de Candelaria, Santa Cruz de Tenerife, Spain.

Elena Ramos-Trujillo, F. Javier González-Paredes, Hilaria González-Acosta, Félix Claverie-Martín, Maria Isabel Luis-Yanes, Víctor M. García -Nieto.

2. División de Nefrología Pediátrica y Laboratorio de Genética Molecular, Hospital Universitario Central de Asturias, Oviedo, Spain.

Fernando Santos, Eliecer Coto, Victoria Álvarez, Enrique García, Helena Gil, Natalia Mejía, Vanessa Loredo, Flor Ángel Ordóñez, Julián Rodríguez.

3. División de Nefrología Pediátrica y Laboratorio de Genética Molecular

Hospital Universitario de Cruces, Bilbao, Spain.

Gema Ariceta, Luis Castaño, Leire Madariaga, Gustavo Pérez de Nanclares Leal, Alejandro García Castaño, Mireia Aguirre Meñica.
